# Accelerated developmental adipogenesis programs adipose tissue dysfunction and cardiometabolic risk in offspring born to dams with metabolic dysfunction

**DOI:** 10.1152/ajpendo.00229.2021

**Published:** 2021-08-30

**Authors:** Anna Mikolajczak, Nada A. Sallam, Radha D. Singh, Taylor B. Scheidl, Emma J. Walsh, Sebastian Larion, Carol Huang, Jennifer A. Thompson

**Affiliations:** ^1^Department of Physiology and Pharmacology, University of Calgary, Calgary, Alberta, Canada; ^2^Department of Pediatrics, University of Calgary, Calgary, Alberta, Canada; ^3^Cumming School of Medicine, University of Calgary, Calgary, Alberta, Canada; ^4^Libin Cardiovascular Institute, Calgary, Alberta, Canada; ^5^Alberta Children’s Hospital Research Institute, Calgary, Alberta, Canada; ^6^Faculty of Pharmacy, Cairo University, Giza, Egypt; ^7^Division of Gastroenterology and Hepatology, Medical University of South Carolina, Charleston, South Carolina

**Keywords:** adipogenesis, developmental programming, lipogenesis, programming

## Abstract

This study determined if a perturbation in in utero adipogenesis leading to later life adipose tissue (AT) dysfunction underlies programming of cardiometabolic risk in offspring born to dams with metabolic dysfunction. Female mice heterozygous for the leptin receptor deficiency (Het*_db_*) had 2.4-fold higher prepregnancy fat mass and in late gestation had higher plasma insulin and triglycerides compared with wild-type (Wt) females (*P* < 0.05). To isolate the role of the intrauterine milieu, wild-type (Wt) offspring from each pregnancy were studied. Differentiation potential in isolated progenitors and cell size distribution analysis revealed accelerated adipogenesis in Wt pups born to Het*_db_* dams, accompanied by a higher accumulation of neonatal fat mass. In adulthood, whole body fat mass by NMR was higher in male (69%) and female (20%) Wt offspring born to Het*_db_* versus Wt pregnancies, along with adipocyte hypertrophy and hyperlipidemia (all *P* < 0.05). Lipidomic analyses by gas chromatography revealed an increased lipogenic index (16:0/18:2n6) after high-fat/fructose diet (HFFD). Postprandial insulin, ADIPO-IR, and ex vivo AT lipolytic responses to isoproterenol were all higher in Wt offspring born to Het*_db_* dams (*P* < 0.05). Intrauterine metabolic stimuli may direct a greater proportion of progenitors toward terminal differentiation, thereby predisposing to hypertrophy-induced adipocyte dysfunction.

**NEW & NOTEWORTHY** This study reveals that accelerated adipogenesis during the perinatal window of adipose tissue development predisposes to later life hypertrophic adipocyte dysfunction, thereby compromising the buffering function of the subcutaneous depot.

## Graphical abstract

**Figure:**
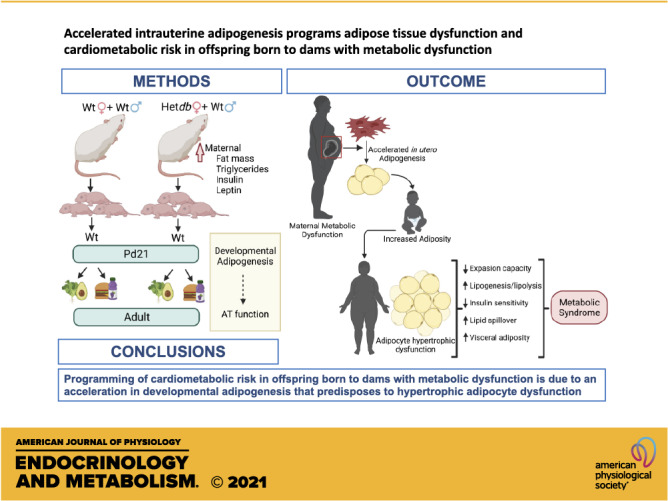

## INTRODUCTION

Since the 1970s, obesity rates among women of reproductive age have more than tripled, reaching over 50% in certain sociodemographic groups (1, 2). Prepregnancy obesity and hypertriglyceridemia are strong predictors of gestational diabetes (GDM), which now occurs in 18% of pregnancies worldwide and up to 25% in certain North American populations (3, 4). These trends have made maternal metabolic disorders the most common complications of pregnancy. Babies born to mothers with obesity or diabetes during pregnancy are more likely to develop cardiometabolic risk factors such as obesity and insulin resistance, often before reaching adulthood (5–7). Thus, in utero transmission may be a critical driver of metabolic disease and its declining age of onset. Although both human and animal studies have established an association between maternal metabolic dysfunction and cardiometabolic risk in the offspring, the underlying developmental mechanisms remain incompletely understood.

Macrosomia primarily due to high fat mass is the most common fetal consequence of pregnancies complicated by maternal obesity or GDM (8, 9). This acceleration in fat mass accumulation during the perinatal window of adipose tissue development may be a key pathogenic factor in later life development of insulin resistance. Lineage tracing studies in mice reveal that although maturation of visceral depots is predominately a postnatal event, adipogenesis in subcutaneous depots starts earlier in fetal life (10). Adipogenesis is the process by which mesenchymal stem cells commit to the adipocyte lineage by differentiating into preadipocytes, followed by terminal differentiation to lipid-storing adipocytes. Proliferation and differentiation of adipocyte progenitors in subcutaneous depots peak in the perinatal period, stabilize in the second week of postnatal life, and peak again in early puberty (10, 11). Thereafter, adipocyte numbers remain constant, but mature depots retain a residual population of committed preadipocytes that support normal adipocyte turnover and play a critical role in the buffering of lipid flux (12). Thus, adipose tissue function in adulthood may be programmed during the critical perinatal period of adipogenesis.

The subcutaneous adipose tissue (SAT) depot, which makes up 85% of total fat mass, serves as the body’s “metabolic sink” that stores and sequesters excess energy (13). When hypertrophic growth of adipocytes reaches a defined limit of expansion, the recruitment of resident preadipocytes for differentiation allows further storage of lipids and maintenance of metabolic health. Thus, a failure in SAT expansion leads to hypertrophic adipocyte dysfunction characterized by engorged adipocytes that are prolipogenic, hypoxic, fibrotic, and resistant to the antilipolytic effects of insulin. This in turn results in lipid spillover and an accumulation of fat in visceral depots as well as nonadipose organs including the liver and blood vessels (14–16). A growing body of evidence reveals that SAT dysfunction precipitates obesity-associated development of cardiometabolic disease. Herein, we demonstrate that programming of SAT dysfunction due to accelerated adipogenesis during the perinatal window of SAT development underlies heightened risk in offspring born to dams that are metabolically compromised.

## MATERIALS AND METHODS

### Animal Model

All experimental protocols were approved by the University of Calgary Animal Care Committee (AC17-0149) and conducted in accordance with guidelines by the Canadian Council on Animal Care Ethics. Obese female mice homozygous for the leptin receptor deficiency are infertile. Thus, to study a consistent and reproducible level of metabolic dysfunction in fertile females, we used heterozygous mice. Female mice heterozygous (Het*_db_*) for the leptin receptor mutation (Jackson Laboratory, strain 000697) and C57BL/6J wild-type control (Wt) females (Jackson Laboratory, strain 000664) were purchased. At 14 wk of age, virgin Het and Wt females were evaluated for body weight and body composition using TD-NMR (LF90II, Bruker) and subsequently mated with C57BL/6J Wt males. Pregnancy was confirmed by the presence of a copulatory plug. Plasma was collected from pregnant dams euthanized on *gestational day 17* (*Gd17*) and evaluated for lipids, insulin, and leptin using colorimetric assays (ALPCO). Pups that delivered spontaneously were weaned on *postnatal day 21* (*Pd21*) and genotyped. Only Wt offspring from each pregnancy were studied to exclude effects of offspring genotype. At 7  wk of age, offspring were fed a diet containing 45% kcal fat and 35% kcal fructose (D08040105I, Research Diets) or a control diet containing 10% kcal fat (D12450KI, Research Diets) until the day of euthanasia.

### Adiposity, Insulin Sensitivity, and Plasma Profiles

Whole body fat and lean body mass were determined by TD-NMR (LF90II, Bruker). Cell size distribution in hematoxylin-eosin (H&E)-stained sections of iSAT captured on a Thorlabs Tide Whole slide-scanning microscope was quantified using Adiposoft Software (Image J). After a 6-h fast, insulin sensitivity was assessed by injecting 0.5 IU/kg insulin (IP) and sampling blood glucose from the tail at baseline and at 15, 30, 45, 60, and 90-min postinjection. At euthanasia, trunk blood was collected immediately upon decapitation with a heparinized syringe and plasma frozen for later analyses. Colorimetric or fluorescent assays were used to assess plasma concentrations of triglycerides (ALPCO), nonesterified free fatty acids (ALPCO), total cholesterol (ALPCO), and Resistin (Abcam).

### Gas Chromatographic Analysis of Fatty Acids

Plasma samples were sent to the Vanderbilt University Medical Center Lipid Lab for identification and quantification of fatty acids in lipid. Briefly, lipids extracted using the Folch-method were filtered and recovered in the chloroform phase (17). Individual lipid classes were separated by thin layer chromatography, visualized by rhodamine 6G, and methylated. Methylated fatty acids were extracted and identified by comparing the retention times to known standards. Odd chain fatty acid standards were used for lipid quantification. The desaturation index, reflecting the biosynthesis of substrates for incorporation into triglycerides and cholesterol, was calculated by the ratio of precursors [palmitic acid (16:0), stearic acid (18:0)] to products [palmitoleic (16:1n-7), oleic acid (18:1n-9)] (18–20). The lipogenic index, reflecting the rate of de novo lipogenesis, was calculated as the ratio of palmitic acid (16:0) to linoleic acid (18:2n6) (20)

### In Vivo and Ex Vivo AT Function

Fluorescent ELISAs (Abcam) were used to measure plasma concentrations of free fatty acid (FFA) (21) and insulin in plasma collected from animals in the postprandial state, according to the manufacturer’s instructions. The AT insulin resistance index (ADIPO-IR) was calculated as insulin (mmol/L) × FFA (pmol/L) (22). Inguinal subcutaneous adipose tissue (iSAT) and gonadal white adipose tissue (gWAT) were aseptically dissected, cut into 50 mg pieces, and placed in a 24-well plate along with medium M199 containing 1% penicillin/streptomycin and fetal bovine serum. On the following day, medium was replaced and treated with isoprenaline (10^−6^ M) for 90 min. The rate of lipolysis was assessed by measuring glycerol output in the medium using an ELISA (Abcam). Glycerol was normalized to protein content of the explants using a Pierce BCA Protein Assay kit. 

### Differentiation Capacity of Adipocyte Progenitors

Under sterile conditions, the iSAT was excised, minced, and enzymatically digested with collagenase in 1 mg/mL Collagenase I in digestion buffer (HBSS, 100 mM HEPES, 1.5% BSA). After digestion, the solution was filtered, centrifuged at 500 *g* for 10 min, and subsequently treated with red blood cell lysis buffer. The isolated stromal vascular fraction (SVF) was filtered through a 70-µm cell strainer and plated in preadipocyte growth medium (Cell Applications Inc.). Isolated adipocyte progenitors were studied for differentiation capacity after one passage. Cells were treated with differentiation medium (Cell Applications Inc.) 48 h after contact inhibition and subsequently maintained in maintenance medium (Cell Applications Inc.). Cells were collected on *days 2*, *4*, and *7* after induction of differentiation. Cells were fixed in 4% paraformaldehyde for 1 h, washed and rinsed in 60% isopropyl alcohol for 30 s before being stained with Oil Red O solution diluted from stock solution [0.4% Oil Red O (Sigma-Aldrich) in isopropyl alcohol]. After thorough washing, stained cells were imaged with a Nikon Eclipse Ts2 microscope and the image processed with NIS-Elements D 5.11.00. Subsequently, Oil Red O staining was quantified with a spectrophotometer (SpectraMax M2 plate reader) after incubating the cells with lysis buffer (4% IGEPAL in isopropyl alcohol) for 10 min. Triplicate readings were taken for each of the three wells plated per animal. Some wells were stained with 2 µM BODIPY (Thermo Fisher Scientific) for 15 min at 37°C and imaged.

### Gene Expression

Total RNA was extracted using an RNA extraction kit (Qiagen), assessed for RNA integrity using a TapeStation RNA Assay, and quantified with an N50 Nanophotometer (Implen Inc.). Complementary DNA was synthesized from 2-µg purified RNA using the High Capacity cDNA Reverse Transcription kit (Applied Biosystems). Primers used for analysis (Table 1) were chosen among three primer pairs designed with BLAST and tested for efficiency and specificity. Quantitative real-time PCR was performed with Powerup SYBR green master mix (Applied Biosystems) on a QuantStudio 5 Real-time PCR System (Applied Biosystems). Amplification was performed in triplicate and the expression of individual target genes calculated relative to β-actin and expressed as fold change relative to the control group using the 2^−ΔΔCt^ method.

**Table 1.** Primer pairs

Gene	Sequence		Accession
*SREBF1*	Forward Reverse	AACTTTTCCTTAACGTGGGC	NM_011480.4
		CATGTCTTCGATGTCGTTCA
*ZFP423*	Forward Reverse	AACAAAGTTTCCGAGAGGC	NM_033327.2
		CCCTCTTCAACTTTCACCGA
*C/EBPβ*	Forward Reverse	CAACACACGTGTAACTGTCA	NM_001287738.1
		CGAAACGGAAAAGGTTCTCA
*FABP4*	Forward Reverse	GACAAGCTGGTGGTGGAATGTG	NM_024406.3
		CCATCCAGGCCTCTTCCTTTG
*mTOR*	Forward Reverse	TATCCGCTACTGTGTCTTGG	NM_020009.2
		TGCTCCTTGATTCTCCCAAT
*LXR*	Forward Reverse	AGAGAGATGGAACTAGACCG	AJ132599.1
		TAGCTTCCCCACCAGACTAA
*β-Actin*	Forward Reverse	GATCAAGATCATTGCTCCTCCT	XM_030254057.1
		GTAACAGTCCGCCTAGAAGC

### Protein Expression

Protein was extracted in RIPA Lysis buffer (Invitrogen) together with a protease and phosphatase inhibitor cocktail (Invitrogen) and quantified with a Pierce BCA Protein Assay kit. Extracted protein was mixed with NuPAGE lithium dodecyl sulfate sample buffer (Invitrogen) and reducing agent, heated for 10 min at 85°C, and separated in a precast NuPAGE 4%–12% Bis-Tris polyacrylamide gel (Invitrogen) by SDS-PAGE electrophoresis. Separated proteins were transferred to Amersham Hybond PVDF membranes (GE Healthcare Life Sciences) at 100 V for 2 h. Following transfer, membranes were washed and blocked for 1 h and then probed with primary antibodies preincubated in the blocking agent. Primary antibodies used were: SREBP1 (Novus Biologicals; 1:500), FABP4 (Cell Signaling; 1:400), Adiponectin (Abcam; 1:1,000), C/EBPβ (Cell Signaling; 1:500), and C/EBPα (Cell Signaling; 1:500). After washing and incubation with the secondary antibody, immunoreactivity was visualized by chemiluminescence and densitometry performed with an iBright CL1500 Imaging system (Applied Biosystems).

### Statistical Analysis

Statistical analyses were performed using GraphPad Prism 7. Differences in maternal, neonatal, or adult data between Wt and Het*_db_* groups were assessed using an unpaired Student’s *t* test. Differences in cell size frequency as well as diet and in utero effects in adult offspring were assessed with a two-way ANOVA followed by Sidak’s multiple comparisons test. Body weight and composition were recorded for each offspring; all other experiments included one offspring per dam. The data are expressed as means ± SE and a *P* value < 0.05 considered to be statistically significant.

## RESULTS

### Metabolic Dysfunction in Pregnant Mice Heterozygous for Leptin Receptor Deficiency

The metabolic profile of pregnant and nonpregnant female mice purchased from Jackson is shown in Supplemental Fig. S1 (see https://doi.org/10.6084/m9.figshare.14798748). Immediately before mating, the body weight of virgin Het*_db_* females was 29% higher than age-matched Wt females (*P* < 0.0001). Whole body composition analysis by NMR spectroscopy revealed that prepregnancy fat mass was 2.4-fold higher and lean body mass 13% lower in Het*_db_* versus Wt females (*P* < 0.0001). On Gd17, pregnant Het*_db_* dams had higher plasma insulin (Wt: 0.017 ± 0.0049 ng/dL vs. Het*_db_*: 0.257 ± 0.0448 ng/dL, *P* = 0.0002), triglycerides (Wt: 49.2 ± 3.40 mg/dL vs. Het*_db_*: 76.4 ± 11.06 mg/dL, *P* = 0.047), and leptin (Wt: 2547 ± 244.9 pg/mL vs. Het*_db_*: 7495 ± 1630 pg/mL, *P* = 0.0045). No differences in maternal plasma nonesterified FFA or cholesterol were found (data not shown). Therefore, the Het*_db_* female recapitulates central features of human pregnancies complicated by maternal obesity and GDM, including high prepregnancy adiposity, hyperinsulinemia, hypertriglyceridemia, and hyperleptinemia.

### Maternal Metabolic Dysfunction Programs Heightened Cardiometabolic Risk in Offspring

**Figure 1.**
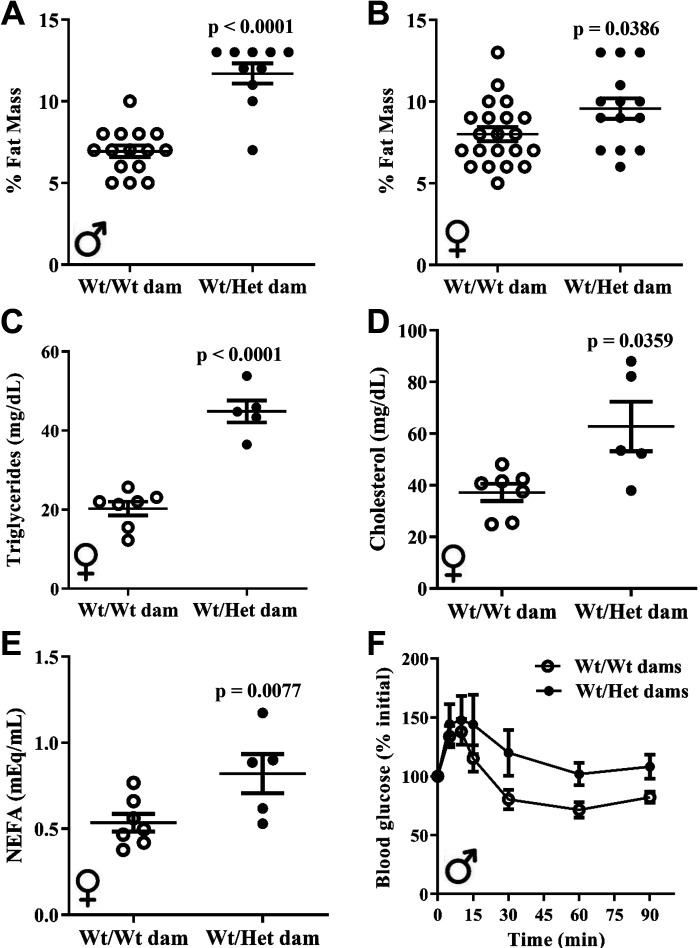
Heightened cardiometabolic risk in offspring born to metabolically compromised dams. In 12-wk-old chow-fed Wt male (*A*) and female (*B*) offspring born to Wt or Het*_db_* pregnancies (Wt/Wt vs. Wt/Het), whole body fat mass was measured by TD-NMR. Fasting plasma triglyceride levels (*C*), total cholesterol (*D*), and nonesterified FFA (*E*) were evaluated in 6-mo-old female offspring using colorimetric assays. Insulin sensitivity (*F*) was assessed after a 6-h fast by injecting 0.5 IU/kg insulin (IP) and sampling blood glucose from the tail (*n* = 6–8).

Cardiometabolic risk factors in offspring exposed to the metabolic perturbations of Het*_db_* pregnancy were studied. At 12 wk of age, whole body fat mass in chow-fed male and female Wt offspring born to Het*_db_* pregnancies was greater by 69% (*P* < 0.0001) and 20% (*P* = 0.038), respectively (Fig. 1, *A* and *B*). There was an increase in fasting plasma triglyceride levels, total cholesterol, and FFA in 6-mo-old female Wt offspring born to Het*_db_* dams (Fig. 1, *C*–*E*). Plasma lipid concentrations were not measured in chow-fed male offspring. The drop in blood glucose levels in response to an insulin injection was blunted in male offspring born to Het*_db_* dams (Fig. 1*F*). Female offspring displayed no differences in insulin sensitivity, as shown in Supplemental Fig. S2 (see https://doi.org/10.6084/m9.figshare.16637659.v1). These findings show that the metabolic perturbations of Het*_db_* pregnancy reproduce the programming of cardiometabolic risk factors reported in human offspring exposed to mothers with obesity or GDM (6, 7).

### Cardiometabolic Risk Accompanied by Prolipogenic Lipidomic Profile

**Figure 2.**
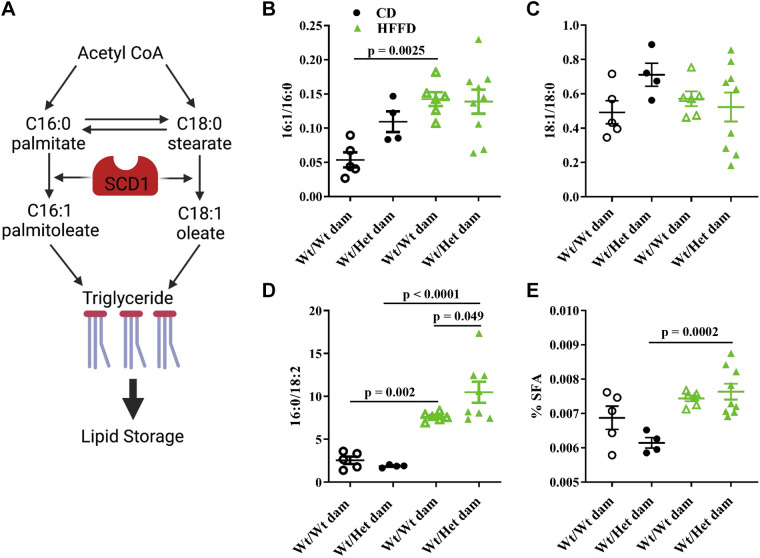
Increased activity of lipogenic enzymes. Lipidomic analysis was carried out by thin layer gas chromatography in lipids extracted from plasma collected from adolescent male offspring born to Wt or Het*_db_* dams (Wt/Wt vs. Wt/Het) that were fed a control diet (CD) or a high-fat/fructose diet (HFFD). The lipogenic enzyme, stearoyl CoA desaturase (SCD1) catalyzes the conversion of saturated fatty acids (SFA), palmitate and stearate, to monounsaturated fatty acids (palmitoleate and oleate) that are incorporated into complex lipids like triglycerides (*A*). The desaturation index was calculated by the ratio of palmitic acid (*B*) and stearic acid (*C*) to their monounsaturated products. The lipogenic index (*D*), reflecting the rate of de novo lipogenesis, was calculated as the ratio of palmitic acid (16:0) to linoleic acid (18:2n6). The percentage of total saturated fatty acids (SFA) relative to all lipid species (*E*) was calculated.

Lipidomic analysis of plasma extracts was carried out in adolescent (10 wk old) offspring fed a control (CD) or high-fat/fructose diet (HFFD). Lipogenesis involves the desaturation of saturated fatty acids to monounsaturated for incorporation into complex lipids, such as triglycerides and cholesterol (Fig. 2*A*). This reaction is catalyzed by the lipogenic enzyme, stearoyl CoA desaturase enzyme 1 (SCD1). The activity of SCD1, otherwise known as the desaturation index, can be inferred by the ratio of products (palmitoleic, 16:1 and oleic, 18:1) to precursors (palmitic acid, 16:0 and stearic acid, 18:0) (23). HFFD led to an increase in the ratio of palmitoleic to palmitic acid in offspring born to control dams, whereas HFFD led to no further increases in 16:1/16:0 in Het*_db_* offspring (Fig. 2*B*). A similar pattern across groups was observed in the ratio of oleic acid to stearic acid (Fig. 2*C*). The lipogenic index, reflecting the enrichment of triglycerides in palmitate (16:0) and depletion in diet-derived linoleic acid (18:2n-9), was increased in both groups of offspring with HFFD feeding, a response that was significantly greater in Wt offspring born to Het*_db_* pregnancy (Fig. 2*D*). The % of saturated fatty acids (SFA) in lipid extracts was increased with HFFD in only the Het*_db_* offspring (Fig. 2*E*). These data show that offspring born to Het*_db_* pregnancy exhibit a prolipogenic metabolic profile, which is associated with hyperlipidemia, ectopic lipid deposition, and insulin resistance.

### Adipocyte Hypertrophy, Lipid Spillover, and Impaired Antilipolytic Effects of Insulin

**Figure 3.**
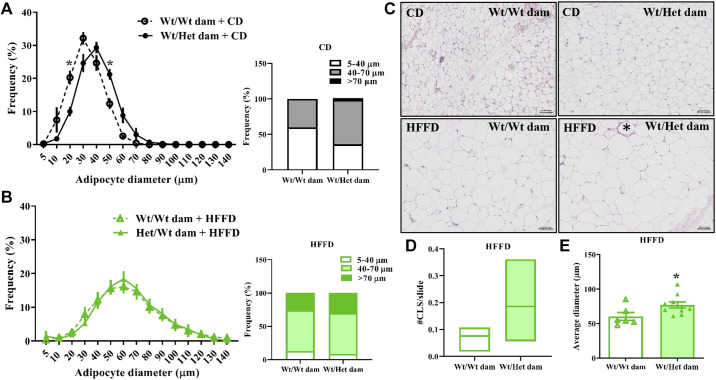
Adipocyte hypertrophic dysfunction. Cell size distribution calculated with AdipoSoft (Image J) in sections of iSAT of Wt male offspring born to Wt or Het*_db_* dams (Wt/Wt vs. Wt/Het) fed a control diet (CD) (*A*) or high-fat/fructose diet (HFFD) (*B*). Representative sections of iSAT (*C*) in CD or HFFD-fed male offspring (* denotes crown-like structure). Number of crown-like structures (CLS) per slide was quantified in iSAT sections of HFFD-fed male offspring (*D*). *n* = 4–5. Hypertrophy of visceral adipocytes after HFFD was determined by quantifying average diameter in H&E sections of gonadal fat (*E*). *P < 0.05. H&E, hematoxylin-eosin.

**Figure 4.**
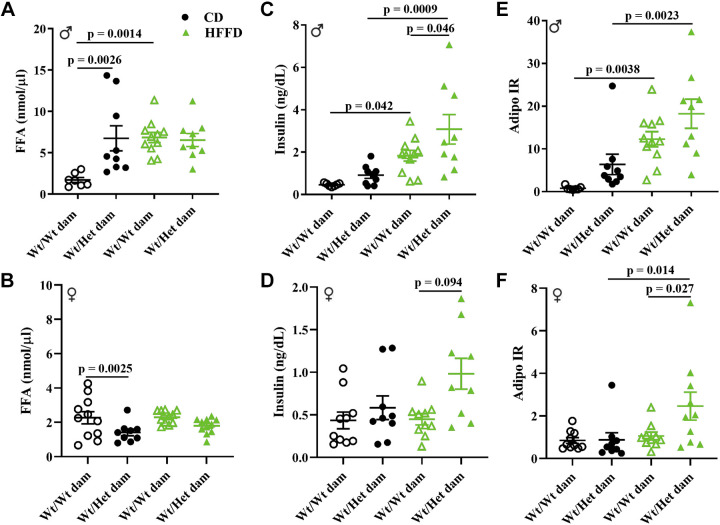
Lipid spillover and suppressed antilipolytic effects of insulin. Postprandial levels of FFA in male (*A*) and female (*B*) Wt offspring born to Wt or Het*_db_* dams (Wt/Wt vs. Wt/Het). Postprandial insulin levels in male (*C*) and female (*D*) offspring were measured, and the ADIPO-IR was calculated as insulin × FFA for male (*E*) and female (*F*) offspring.

**Figure 5.**
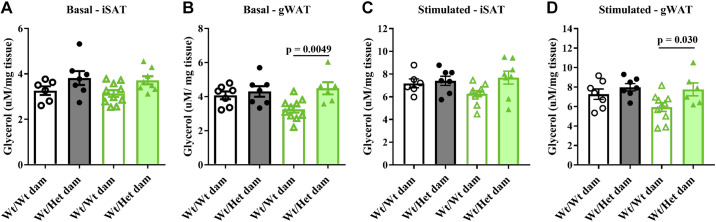
Higher basal and stimulated lipolysis in visceral SAT explants. In explants of inguinal (iSAT) or gonadal white adipose tissue (gWAT) isolated from male Wt offspring born to Wt or Het*_db_* dams (Wt/Wt vs. Wt/Het), glycerol output, reflecting the rate of lipolysis, was measured at baseline (*A* and *B*) or after stimulation with isoproterenol (*C* and *D*).

Engorged adipocytes that have reached their expansion capacity become inflamed, insulin resistant, and fail to buffer excess energy, leading to lipid spillover and ectopic lipid deposition (12, 24). In the iSAT of CD-fed adult offspring born to Het*_db_* dams, there was a lower abundance of small-diameter adipocytes (10–40 µm) and a greater frequency of large adipocytes (Fig. 3*A*). HFFD led to a markedly greater increase in large adipocytes and decrease in small-diameter adipocytes in offspring born to Wt pregnancies, abolishing the differences between the two groups of offspring (Fig. 3*B*). These data suggest that offspring born to Het*_db_* pregnancy had lower expansion reserves, predisposing them to diet-induced hypertrophic dysfunction. Inflammation in hypertrophied adipocytes leads to the formation of crown-like structures (CLS), which tended to be present in higher amounts in iSAT of HFFD offspring from Het*_db_* dams (Fig. 3, *C* and *D*). Adipocytes in gonadal AT were more hypertrophied after HFFD feeding in offspring from Het*_db_* dams (Fig. 3*E*), suggesting lipid spillover into the circulation. In 22-wk-old CD males, postprandial FFA was higher in those born to Het*_db_* versus Wt pregnancies (Fig. 4*A*). HFFD-feeding led to an increase in postprandial FFA in Wt males born to control pregnancies, whereas no further diet-induced increases in FFA were observed in males born to Het*_db_* dams (Fig. 4*A*). Female offspring were protected from increases in FFA due to both diet and in utero effects (Fig. 4*B*). Male offspring born to Het*_db_* dams had higher postprandial insulin levels when fed either a CD or HFFD and insulin was also higher in HFFD-fed females born to Het*_db_* versus Wt dams (Fig. 4*C*). Insulin sensitivity in adipose tissue was assessed by calculating the ADIPO-IR. These data reveal that FFA levels in relation to insulin were increased in response to HFFD-feeding in male offspring born to either control or Het*_db_* pregnancies (Fig. 4*E*). In female offspring, the ADIPO-IR increased in response to HFFD in only Het*_db_* offspring and there was a significant difference between HFFD females born to Het*_db_* versus Wt dams (Fig. 4*F*). Lipolysis in adipose tissue explants collected from male offspring was determined by measuring glycerol output at baseline and after stimulation with isoproterenol. In gonadal AT (gWAT), basal and stimulated glycerol output was higher in HFFD offspring born to Het*_db_* versus Wt pregnancies (Fig. 5, *B* and *D*). No differences in lipolysis were observed in iSAT (Fig. 5, *A* and *C*). Overall, these data show that HFFD unmasks a predisposition to dysregulated lipolysis and lipid spillover in offspring born to Het*_db_* pregnancy.

### Later Life Perturbations in Lipid Homeostasis Linked to Accelerated In Utero Adipogenesis

**Figure 6.**
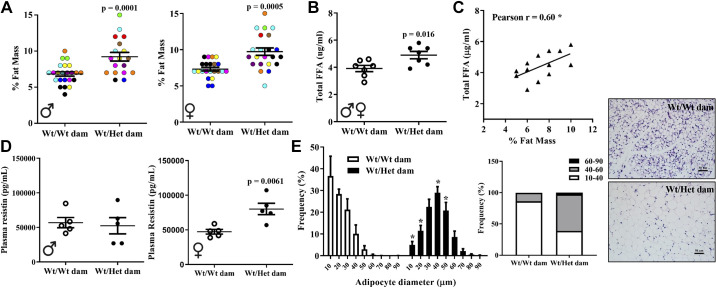
Greater fat mass and accelerated adipogenesis in pups exposed to a metabolically adverse in utero environment. Percent fat mass (*A*) was measured in 3-wk-old Wt pups born to Wt or Het*_db_* dams (Wt/Wt vs. Wt/Het) using a TD-NMR whole body composition analyzer (all littermates are included; litters are color-coded). Plasma levels of total FFA were measured in *Pd21* plasma using thin layer chromatography (*B*) and correlated to percent fat mass (*C*). Resistin levels were determined in plasma using an ELISA (*D*). Cell size distribution was examined using Adiposoft (ImageJ) in H&E-stained sections of Pd21 SAT (*E*) (*n* = 6–8). *P < 0.05 Wt/Wt dam vs. Wt/Het dam. H&E, hematoxylin-eosin.

**Figure 7.**
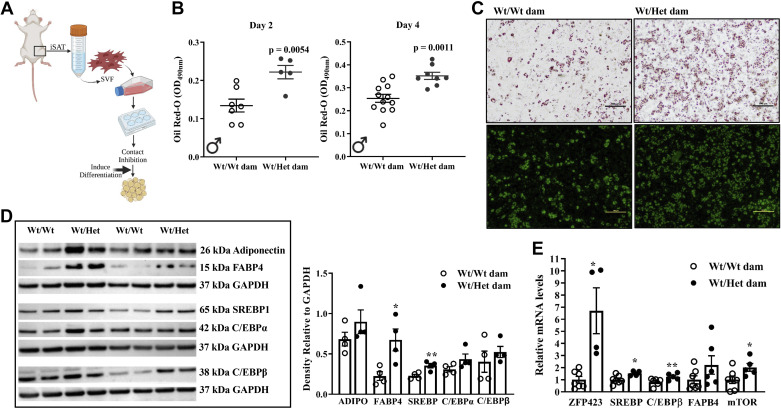
Higher adipogenic potential in progenitors isolated from neonates exposed to metabolic dysfunction in utero. Adipocyte progenitors were cultured from the stromal vascular fraction (SVF) isolated from iSAT of 3-wk-old Wt offspring born to Wt or Het*_db_* dams and induced to differentiate 48 h after contact inhibition (*A*). Quantification of lipid droplet formation by optical density of Oil Red O staining (*B*) and representative images of cells stained with Oil Red O or BODIPY on *day 4* of differentiation (*C*) in progenitors isolated from the SVF of Pd21 iSAT. Protein expression (*D*) and mRNA expression (*E*) of adipogenic and lipogenic mediators on D2 of differentiation were determined. *P < 0.05 or **P < 0.001: Wt/Wt dam vs. Wt/Het dam.

To test whether iSAT dysfunction is secondary to postnatal development of obesity or a direct manifestation of a developmental perturbation, we examined adipogenesis in Pd21 offspring. Het*_db_* pregnancy was associated with a 32% and 33% higher whole body fat mass in male and female Wt pups, respectively, as measured by NMR (Fig. 6*A*). Body weight was higher in female (8.0 ± 0.21 g vs. 10.0 ± 0.52g, *P* = 0.0007) and male (8.4 ± 0.22g vs. 10.4 ± 0.80g, *P* = 0.0059) pups born to Het*_db_* dams. Circulating FFA, which derive predominately from AT, was higher in Wt pups born to Het*_db_* pregnancies and positively correlated with the degree of adiposity (Fig. 6, *B* and *C*). Resistin, an adipokine secreted from mature adipocytes, was increased in the plasma of female pups born to Het*_db_* pregnancies, but unchanged in male pups (Fig. 6*D*). The distribution of adipocyte diameter in iSAT of pups born to Het*_db_* dams was shifted toward large, mature adipocytes (Fig. 6*E*). Adipocyte size distribution cannot differentiate between differences in lipid deposition or adipogenesis. Thus, differentiation capacity was assessed in adipocyte progenitors isolated from the SVF of iSAT (Fig. 7*A*). Differentiation efficiency was greater in adipocyte progenitors isolated from the iSAT of pups born to Het*_db_* dams, as determined by Oil Red O and BODIPY staining of lipid droplets (Fig. 7, *B* and *C*). On *day 2* of differentiation, the protein or mRNA expression of early mediators of adipogenesis and lipogenesis (ZFP423, FABP4, SREBP1, C/EBPβ, mTOR) were increased in progenitors isolated from pups born to Het*_db_* pregnancies (Fig. 7, *D* and *E*). Together, these data suggest that accelerated adipogenesis is responsible for the higher accumulation of fetal fat mass that characterizes pregnancies complicated by maternal metabolic disorders.

## DISCUSSION

Children, adolescents, and adults born to mothers with obesity or diabetes during pregnancy are more likely to develop insulin resistance and other components of the metabolic syndrome (5, 7, 25). Although the link between maternal metabolic status and cardiometabolic risk in the offspring is well established, there remains a shallow understanding of the underlying developmental roots.

To model the intrauterine environment created by metabolic complications of pregnancy, we used Het*_db_* female mice, which produced a consistent degree of gestational metabolic dysfunction while retaining fertility. Specifically, our data show that the Het*_db_* female mimics metabolic derangements common to maternal obesity and GDM, including high maternal adiposity, hyperinsulinemia, hypertriglyceridemia, and hyperleptinemia (26, 27). This model reproduced the programming of cardiometabolic risk factors reported in human offspring born to pregnancies complicated by these metabolic conditions. The Het*_db_* female is therefore a valuable tool for probing the developmental origins of cardiometabolic disease in offspring exposed to an abnormal metabolic intrauterine milieu.

The developmental origins of chronic disease are thought to stem from programming of structural and functional deficits owing to aberrations in cellular proliferation and differentiation during critical periods of organ maturation. Given that the most common fetal consequence of maternal metabolic disease is macrosomia predominately due to excess fat mass, later life insulin resistance may arise from a perturbation in adipose tissue development (8, 28). Rapid accretion of subcutaneous fat starts in late gestation and continues into early postnatal life during both human and murine development, although fat mass comprises a much larger portion of birth weight in the human newborn compared with the neonatal rodent. Subcutaneous depots serve as the body’s “metabolic sink” that stores excess energy and prevents spillover of lipids into the circulation and consequent deposition into the secondary visceral depots and nonadipose organs (12, 13).

Lineage tracing studies in mice reveal that adipocyte lineage commitment and differentiation in subcutaneous depots is initiated in the embryonic period between E14 and E18 and continues into the first month of life (10). Seminal work by Graff et al. (29) showed that progenitors committed to the adipocyte lineage before birth are the source of newly formed adipocytes that support hyperplastic AT expansion in adulthood. Therefore, fetal life is a critical window of SAT development that programs its capacity for lipid buffering, an important predictor of metabolic health. Our data reveal that this critical period of cell determination and adipose tissue formation is perturbed in pups exposed to maternal metabolic dysfunction, as evident by higher fat mass and a shift in the distribution of adipocyte size toward larger lipid-laden cells. Stromal vascular cells isolated from the subcutaneous depot of *Pd21* pups differentiated more readily into adipocytes upon stimulation, which may reflect a proadipogenic progenitor phenotype or a greater presence of committed preadipocytes already primed for differentiation before contact inhibition. We propose that a greater number of progenitors directed towards terminal differentiation predisposes to later life hypertrophy-induced adipose tissue dysfunction.

The proadipogenic effect of maternal metabolic dysfunction revealed in the current study is corroborated by studies using murine models of diet-induced maternal obesity; however, there are key differences worthy of consideration. Yang et al. (30) reported higher adipogenic potential in cultured embryonic fibroblasts isolated from E14.5 fetuses from C57BL6/J dams fed a high-fat diet (45%) for 8 wk. At weaning, lipid droplet formation was increased in adipocyte progenitors isolated from retroperitoneal AT of pups born to female rats fed a high-calorie liquid diet exclusively during gestation (31), and in progenitors isolated from epididymal fat of pups born to C57BL6/J dams fed a high-fat (45%) diet for 8 wk preconception and throughout pregnancy and lactation (32). These studies along with others in the literature focus on developmental adipogenesis in visceral depots, although the latter study reported a shift in cell size distribution in iSAT similar to our findings. We chose subcutaneous fat as the focus of our study because it is the primary depot responsible for lipid buffering in adulthood and the preadipocyte reserve critical to this buffering function is established before birth (29). The adipocyte expandability hypothesis posits that hyperlipidemia and visceral obesity develop secondary to a failure in the expansion capacity of iSAT. In addition, it is important to note that an increase in birth weight or fat mass at weaning is not consistently found in models of diet-induced maternal obesity (30–32). In our own laboratory, we find that high-fat feeding for 8 or 6 wk before pregnancy leads to severe growth restriction and reduced litter size (unpublished data). In contrast, wild-type pups born to Het*_db_* dams had higher body weight and fat mass at weaning; although, we cannot rule out the possibility that the lactation period or high levels of maternal leptin played a role in the accumulation of fat mass and hypertrophy of adipocytes. Nevertheless, this model reproduces early life accumulation of fat mass, the most common outcome of human babies born to mothers with obesity or diabetes.

The intrauterine signals that stimulate fetal adipogenesis are yet identified; however, insulin is a likely candidate given its potent effect on in vitro adipogenesis and its critical role in driving fetal growth. Although the placenta is impermeable to insulin, maternal glucose freely crosses the placental barrier and stimulates insulin production by the fetal pancreas, which becomes responsive to glucose later in gestation. Fetal hyperinsulinemia is considered the major culprit for macrosomia in pregnancies complicated by maternal diabetes (33). Maternal lipids, which are increased in both obesity and diabetes, have also been implicated in the fetal overgrowth that is common to pregnancies complicated by these metabolic disorders (34). The fetus is exposed to maternal fatty acids that readily cross the placenta or are released from triglycerides hydrolyzed by placental enzymes. The Het*_db_* model of maternal metabolic dysfunction is unable to differentiate the contribution of specific metabolic stimuli to accelerated fetal adipogenesis, but it is possible that insulin, lipids, or both play a role.

The higher frequency of large adipocytes observed in the iSAT of pups born to dams with metabolic dysfunction persisted into adulthood, at which time diet-induced hypertrophy was markedly attenuated. These data suggest that accelerated in utero adipogenesis limits later life lipid storing capacity, thereby lowering the threshold at which weight gain leads to lipid spillover and hypertrophy-induced adipocyte dysfunction. AT dysfunction is characterized by an imbalance in lipid metabolism with reductions in β-oxidation of FFA and increases in lipogenesis and lipolysis. We used lipidomic analysis to assess the activity of lipogenic enzymes, as paradoxical decreases in the expression of these enzymes have been reported in adverse metabolic states associated with increased lipogenesis (35, 36). The lipogenic index calculated as the ratio of palmitic (16:0) to linoleic (18:2n6) was increased in intrauterine-exposed male offspring. This ratio reflects the rate of de novo lipogenesis (DNL), which is low under normal conditions, but increases in obesity and predicts insulin resistance, dyslipidemia, and ectopic lipid deposition in the liver (21, 37). Also calculated was the desaturation index (16:1/16:0) reflecting the addition of a double bond at the Δ-9 position of SFA by the enzyme SCD1, a rate limiting step in the synthesis of complex lipids like triglycerides and cholesterol. This ratio was markedly increased by HFF feeding in offspring of normal pregnancies, likely reflecting higher lipid storage. In offspring born to metabolically adverse pregnancies, the desaturation index was high with control diet and no further increases were observed after HFFD feeding. In CD-fed males, postprandial FFA levels and ADIPO-IR were higher, although the latter did not reach significance, in those born to abnormal pregnancy. In females, differences in ADIPO-IR between the two groups of offspring were only apparent after HFFD feeding. The lipolytic effects of isoproterenol were amplified in visceral but not subcutaneous explants isolated from exposed offspring. This finding may be explained by the more exaggerated lipolytic responses to β-adrenergic agonists in visceral depots (38), despite SAT being the main contributor to plasma FFA. Overall, our findings provide evidence that an acceleration in early life adipogenesis and the accumulation of fat mass predispose to later life iSAT dysfunction and its metabolic consequences.

### Conclusions

In this study, we identified a developmental perturbation linking metabolic complications of pregnancy to cardiometabolic risk in the offspring. Components of the metabolic syndrome in exposed offspring were accompanied by adipocyte hypertrophy and abnormalities in lipid metabolism indicative of a failure in the buffering function of AT. Our data suggest that this breakdown in lipid homeostasis traces directly to intrauterine stimuli that accelerate differentiation of adipocyte progenitors in developing iSAT, thereby lowering later life reserve capacity for further lipid storage.

## SUPPLEMENTAL DATA

Supplemental Fig. S1: https://doi.org/10.6084/m9.figshare.14798748.

Supplemental Fig. S2: https://doi.org/10.6084/m9.figshare.16637659.v1.

## GRANTS

This work was funded by grants held by J.A.T from the Canadian Institutes for Health Research (CIHR, 162452), the Heart and Stroke Foundation of Canada (HSFC, G-19–0026536), the National Institutes of Health (NIH: NIH K99/R00 1K99HD087527-01), and the Canadian Foundation for Innovation (CFI). J.A.T is also funded by the Libin Cardiovascular Institute. A.M. was supported by a scholarship from the Alberta Children’s Hospital Research Institute (ACHRI) and R.D.S. was supported by a scholarship from the Libin Cardiovascular Institute.

## DISCLOSURES

No conflicts of interest, financial or otherwise, are declared by the authors.

## AUTHOR CONTRIBUTIONS

C.H. and J.A.T. conceived and designed research; A.M., N.A.S., R.D.S., T.B.S., E.J.W., S.L., and J.A.T. performed experiments; A.M., N.A.S., R.D.S., T.B.S., E.J.W., and J.A.T. analyzed data; A.M., N.A.S., and J.A.T. interpreted results of experiments; A.M., N.A.S., and J.A.T. prepared figures; A.M., T.B.S., and J.A.T. drafted manuscript; A.M., T.B.S., and J.A.T. edited and revised manuscript; A.M., N.A.S., R.D.S., T.B.S., E.J.W., S.L., C.H., and J.A.T. approved final version of manuscript.
